# Can Insomnia in Pregnancy Predict Postpartum Depression? A Longitudinal, Population-Based Study

**DOI:** 10.1371/journal.pone.0094674

**Published:** 2014-04-14

**Authors:** Signe K. Dørheim, Bjørn Bjorvatn, Malin Eberhard-Gran

**Affiliations:** 1 MoodNet Research Group, Division of Psychiatry, Stavanger University Hospital, Stavanger, Norway; 2 Norwegian Competence Center for Sleep Disorders, Haukeland University Hospital, Bergen, Norway; 3 Department of Global Public Health and Primary Care, University of Bergen, Bergen, Norway; 4 Health Services Research Center, Akershus University Hospital, Lørenskog, Norway; 5 Division of Mental Health, Norwegian Institute of Public Health, Oslo, Norway; 6 Institute of Clinical Medicine, Campus Akershus University Hospital, University of Oslo, Oslo, Norway; Catholic University of Sacred Heart of Rome, Italy

## Abstract

**Background:**

Insomnia and depression are strongly interrelated. This study aimed to describe changes in sleep across childbirth, and to evaluate whether insomnia in pregnancy is a predictor of postpartum depression.

**Methods:**

A longitudinal, population-based study was conducted among perinatal women giving birth at Akershus University Hospital, Norway. Women received questionnaires in weeks 17 and 32 of pregnancy and eight weeks postpartum. This paper presents data from 2,088 of 4,662 women with complete data for insomnia and depression in week 32 of pregnancy and eight weeks postpartum. Sleep times, wake-up times and average sleep durations were self-reported. The Bergen Insomnia Scale (BIS) was used to measure insomnia. The Edinburgh Postnatal Depression Scale (EPDS) was used to measure depressive symptoms.

**Results:**

After delivery, sleep duration was reduced by 49 minutes (to 6.5 hours), and mean sleep efficiency was reduced from 84% to 75%. However, self-reported insomnia scores (BIS) improved from 17.2 to 15.4, and the reported prevalence of insomnia decreased from 61.6% to 53.8%. High EPDS scores and anxiety in pregnancy, fear of delivery, previous depression, primiparity, and higher educational level were risk factors for both postpartum insomnia and depression. Insomnia did not predict postpartum depression in women with no prior history of depression, whereas women who recovered from depression had residual insomnia.

**Limitations:**

Depression and insomnia were not verified by clinical interviews. Women with depressive symptoms were less likely to remain in the study.

**Conclusions:**

Although women slept fewer hours at night after delivery compared to during late pregnancy, and reported more nights with nighttime awakenings, their self-reported insomnia scores improved, and the prevalence of insomnia according to the DSM-IV criteria decreased. Insomnia in pregnancy may be a marker for postpartum recurrence of depression among women with previous depression.

## Introduction

Insomnia is both a symptom of depression and a separate disorder that may precede depression [1]. Women sleep poorly during pregnancy and sleep even less in the postpartum period [2], [3]. At the same time, the perinatal period is a time of increased risk for depressive disorders [4]. Insomnia during pregnancy may, therefore, influence the risk of postpartum depression.

Insomnia is defined as repeated difficulty with sleep initiation, duration, consolidation, or quality that occurs despite adequate time and opportunity for sleep, and results in some form of daytime impairment [5]. Among postpartum women, there may not be adequate time and opportunity for sleep; the newborn baby requires attention and will disturb maternal sleep in the first months after delivery [6]. However, postnatal women may be exposed to similar practical challenges with sleep and infant care postpartum, but some may develop larger sleep problems than others or be more vulnerable to these changes. A review of 21 longitudinal studies found that individuals with insomnia had a twofold risk of developing depression and that residual insomnia after recovery from depression was associated with an increased risk of relapse [7]. A large, population-based study from Norway found that not only did depression precede insomnia but also that insomnia preceded depression by many years [8]. Similar results have been found in Finland [9]. Furthermore, treating insomnia in depressed patients leads to a better outcome of depression than treating depression on its own [10]. Several studies suggest that insomnia may be co-morbid with depression, with a shared genetic component and a common final pathway [11].

Cross-sectional studies have found that insomnia and depressive symptoms are conditions associated with each other both before [2], [12] and after delivery [13], [14]. Depression during pregnancy is a risk factor for preterm birth [15] and may affect the physiology of the newborn's brain [16]. Furthermore, maternal depression both during pregnancy and after delivery may affect the infant's cognitive and emotional development [17], [18], and these effects may persist through the childhood and teenage years [19], [20]. Prenatal depression is associated with sleep disturbances in the newborn (less deep sleep and more disorganized sleep) [21], and both prenatal and postnatal depression is associated with infant sleep problems one year after delivery [18], [22]. Poor maternal sleep has been linked to perceived sadness of the infant (perceived by the mother) [23]. Poor sleep may also influence the relationship between the parents of the baby [6], [24], and a poor relationship with the partner is a risk factor for postpartum depression [25]. Longitudinal studies of change in sleep and depression across childbirth are few. One study of 44 low-risk women suggested that change in subjective sleep, more than objective sleep, predicts postpartum mood in the first week postpartum [26]. Marques et al., in a study of 382 women, found that insomnia in pregnancy was a predictor for postpartum depressive symptoms along with negative affect, but insomnia did not remain a risk factor when controlling for lifetime depression [14]. Conversely, Okun et al., in a study of 51 perinatal women with a history of postpartum depression, found that women with *fewer* sleep complaints in late pregnancy had a more rapid recurrence (less than 4 weeks) of postpartum depression, whereas women with sleep problems during pregnancy developed postpartum depression later on [27]. Change in sleep in the postpartum period may predict the development of depression more than changes in hormones do [28]. More information is needed regarding the longitudinal relationship between insomnia and depressive symptoms in the perinatal period.

### Aims of the study

The aims of the study were to describe changes in sleep patterns, insomnia, and depressive symptoms before and after delivery, and to evaluate whether insomnia during pregnancy may predict postpartum depression. The study was done in a large, population-based cohort of pregnant women followed through childbirth until eight weeks after delivery, controlling for a range of known risk factors for perinatal depression.

## Material and Methods

### Study population and design

**Figure 1:**
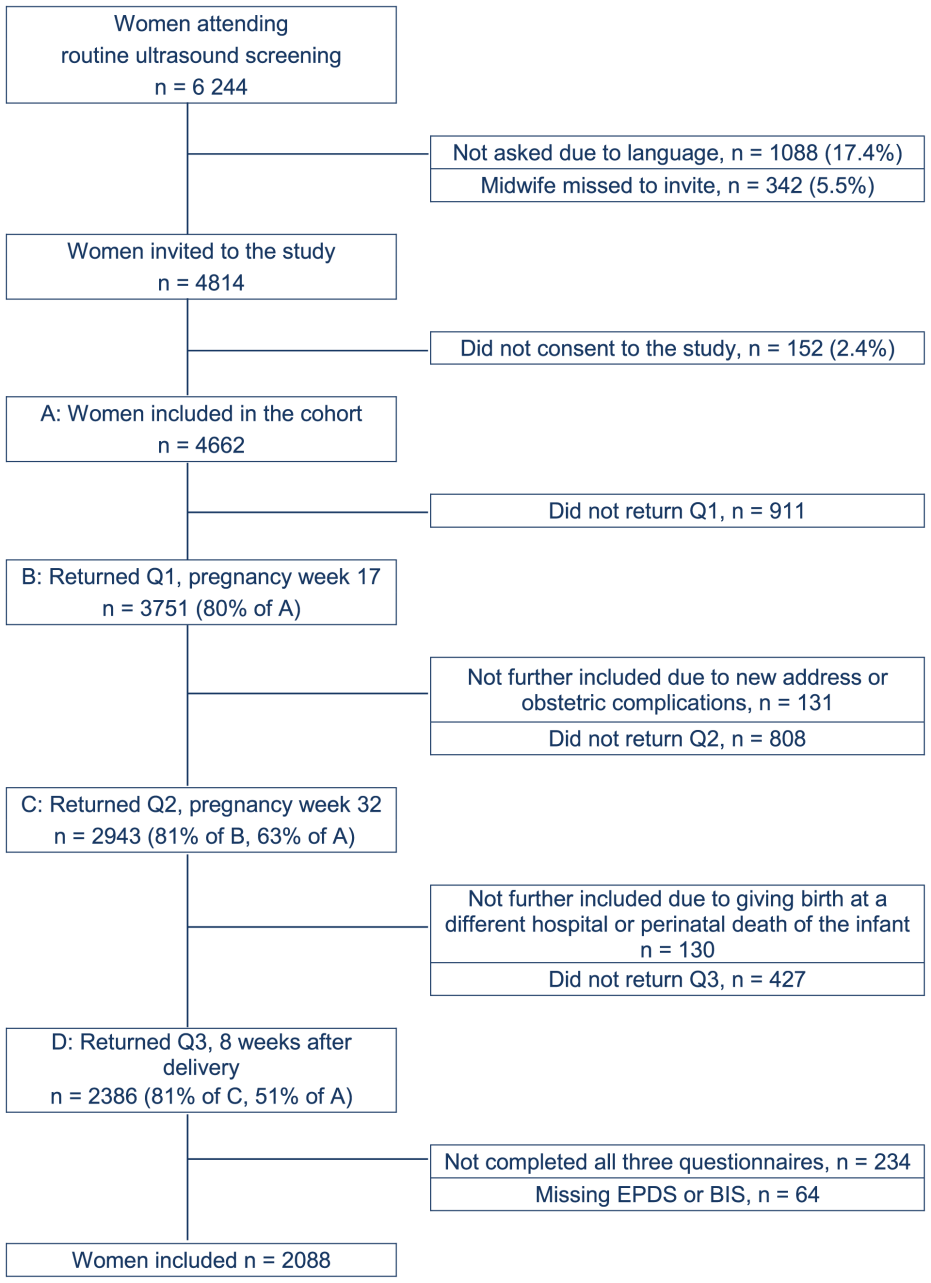
Study flow chart. Legend: EPDS – Edinburgh Postnatal Depression Scale. BIS – Bergen Insomnia Scale.

The Akershus Birth Cohort is a longitudinal questionnaire study targeted at all women giving birth at Akershus University Hospital, Norway. The hospital is located near the capital city of Oslo and serves a population of 350,000 from urban and rural areas. All women scheduled to give birth at the hospital were approached in gestational week 17, when they underwent routine fetal ultrasound. Women were included if they gave consent to participate and were able to complete a questionnaire in Norwegian. Recruitment took place from November 2008 until April 2010. Consenting women were handed a questionnaire at gestational week 17 and, thereafter, received a questionnaire by mail at week 32 of pregnancy and eight weeks after delivery. Information was also retrieved from the birth records at the maternity ward. Figure 1 displays a flowchart of the recruitment and retention of study participants. The current paper examined insomnia and depressive symptoms as measured in week 32 of pregnancy and week eight postpartum. In total, 2,386 women returned the third questionnaire, making the participation rate 51.2% (n = 2386) of the 4,662 women who originally consented to participate, and 81.1% of the women who returned the second questionnaire. The final sample consisted of 2,088 women, as 298 women had missing data for the main outcome variables in one or both questionnaires (insomnia or depressive symptoms).

### Variables

#### Measure of sleep

The Bergen Insomnia Scale (BIS) was used to assess for insomnia (Appendix A) [29]. This questionnaire consists of six items, of which the first four pertain to night factors such as sleep onset delayed more than 30 minutes, waking up for more than 30 minutes during the night, waking up more than 30 minutes earlier than planned, and not feeling adequately rested after sleep. These items correspond to the DSM-IV-TR criterion A for insomnia [30]. The last two items assess level of daytime impairment (affecting work/studies or private life) due to sleepiness and dissatisfaction with sleep, corresponding to the DSM-IV-TR criterion B [30]. Each item is rated on average occurrence from 0 to 7 days per week, giving a possible total sum score from 0 to 42. Women were scored as having insomnia if they had experienced at least one A criterion and one B criterion for three days or more per week during the last month. The BIS has been validated against other self-report scales, as well as polysomnographic data [29]. Cronbach alpha for the BIS was 0.85 in week 32 of pregnancy and 0.74 in week eight postpartum. Three questions from the Pittsburgh Sleep Quality Index (PSQI) [31], [32] were included concerning the average time at which participants went to bed, average wake-up time in the morning, and average total sleep duration per night during the previous month. From these data, habitual sleep efficiency (time asleep divided by total time spent in bed) was calculated. When calculations resulted in sleep efficiencies exceeding 100% (due to women's self-reports), sleep efficiency was recorded as 100%. The women also reported the estimated time their baby was awake during the night. The use of sleep medication was reported for the final 10 weeks of pregnancy.

#### Measure of depressive symptoms

The Edinburgh Postnatal Depression Scale (EPDS) [33], [34] was used to measure depressive symptoms. The EPDS is a 10-item, self-rating questionnaire developed to screen for depression in the postpartum period; it addresses symptoms present during the last seven days. The scale also has good psychometric properties for use during pregnancy [35]. Each question has four possible responses, related to scores from 0 to 3, for a maximum score of 30. Cronbach alpha for the EPDS was 0.85 in late pregnancy and 0.86 in week eight postpartum. A cutoff of 10 or above was found to have good psychometric properties for a diagnosis of depression among Norwegian postpartum women, and this cutoff has also been used in previous studies of pregnant women [36]. Information on previous depression was measured by the Lifetime Major Depression Scale [37]. This scale consists of five questions (concerning sadness, appetite changes, lack of energy, self-blame, and concentration) constructed to measure lifetime history of major depression based on the DSM-IV criteria. Prior depression was defined as having had at least three symptoms at the same time with duration of at least two weeks.

#### Other study factors

The demographic information collected included maternal age, marital status (married or cohabitating versus single/widowed/divorced), number of previous children, and level of education (elementary school, completed high school, or higher education). In pregnancy week 32, we asked about the experience of 10 specific, stressful life events during the last year, as used by previous studies in Norway [13], [38]. Fear of childbirth was measured by the Wijma Delivery Expectancy/Experience Questionnaire version A (W-DEQ) [39], [40]. This is a 33-item, self-assessment scale where each item ranges from 0 to 5, with a total sum ranging from 0 to 165. Serious fear of childbirth was defined as a W-DEQ sum score ≥85. This cutoff is commonly used to distinguish women with a fear of childbirth from women without a fear of childbirth [41]. Symptoms of anxiety were measured by the first 10 items in the Hopkins Symptom Checklist (SCL-25) that comprise the anxiety score (HSCL-A) [42]. Each item ranges from “not at all” (score 1) to “extremely” (score 4). The Norwegian version of SCL-25 has been validated against the ICD-10 criteria for anxiety and depression [43].

### Statistical methods

The data distributions were checked for normality using p-p charts. For numerical data, means and standard deviations were calculated. Differences in means between groups were tested by student's independent t-test, and differences between mean scores before and after delivery for the same women were tested by paired samples t-tests. For categorical data, statistical differences in proportions were examined with chi square. Differences in prevalence of insomnia before and after delivery were tested by McNemar's test. Pearson's correlation was used to test the association between BIS and EPDS. Multivariate linear regressions were used for testing associations with postpartum BIS and EPDS values simultaneously. Factors individually associated with either of these two variables were entered into the model. Parameters with non-normal distribution (EPDS and HSCL) were log transformed before the analyses, and validity of the model was tested by Box's test of equality of covariance matrices and Levene's test for equality of variances. Wilks' Lambda was calculated to test the significance of the multivariate tests, and non-significant variables, except age, were excluded. All analyses were performed in SPSS 18.0 for Windows. The significance level was set to a probability (p) of less than 0.05.

### Ethical considerations

All women asked to participate were given written information explaining the purpose of the study and informed that participation was voluntary. Written informed consent was obtained from all participants, by asking them to sign with full name, address and their date of birth at a separate form. These forms were sent to the project leader, and kept separate from the other data. At the field for date of birth, the following information was given “If you are less than 18 years old, you cannot participate in the study”. In spite of this, three girls aged 17 returned questionnaires, and one had returned all three questionnaires and was included in the present study. This was not discovered until a later phase of the study. The age of majority for health in Norway is 16 years, therefore guardians were not asked for consent, as this would break confidentiality. Since the girl had taken time to complete the questionnaires, and the study did not involve any active interventions, we decided to keep her data in the study. The Regional Committee for Ethics in Medical Research in Norway approved the inclusion of the women aged 17 in the study, provided she now, as adult, gave her consent. Authors did not have any access to identifying information. Anonymization was done before transferring the data from the birth records to the researchers, by personnel at the birth department having this as their job. The forms were sent out to the women's home addresses containing only an anonymous form number, by people not having access to the completed forms returned, and who did not participate in the analyses. Therefore, the data was anonymized upon collection. The study was approved by the Regional Committee for Ethics in Medical Research in Norway, approval number S-08013a.

## Results

The mean age of the postpartum women was 31.5 years (SD 4.7; range 17.4–45.7), and the number of children each woman had (including the new-born) ranged from 1 to 6, median one child, interquartile range 1–2; 11.8% had three or more children. Primipara comprised 50.8% (n = 1,058) of the sample. Two-thirds of the women (n = 1,345; 67.5%) had an education above 12 years of school. Nearly all the women were in stable relationships; only 48 (2.3%) were single. A majority of the women, 70.6% (n = 1,464), were exclusively breastfeeding; 17.8% (n = 370) combined breastfeeding with supplemental formulas, and 241 women (11.6%) did not breastfeed. The response rate among women scoring high for depressive symptoms (EPDS ≥10) in week 32 was 68.0%, significantly lower than among low scorers (74.1%; p = 0.008).

Table 1 displays changes in sleep from late pregnancy to postpartum week eight. Mean sleep duration was significantly reduced after delivery (mean change 49 minutes; p<0.001). The amounts of time women spent in bed after delivery were similar to the time spent in bed in week 32 of pregnancy, resulting in the mean sleep efficiency being reduced from 84.0% to 74.9%, p<0.001. The babies were awake, on average, for one hour and 53 minutes during the night, 25 minutes less than their mothers. Women reported significantly more frequent night-time awakenings in the postpartum period compared to late pregnancy, but the total sum score of insomnia (BIS) improved from 17.2 to 15.4 (p<0.001). The prevalence of insomnia according to the DSM-IV criteria also decreased from 61.5% in late pregnancy to 53.9% after delivery (p<0.001). We did not ask about the present use of sleep medication, but only 12 women (0.6%) reported having used sleep medication during the final months of pregnancy.

**Table 1:** Sleep before and after delivery among 2088 women.

Sleep variable	Week 32 of pregnancy	Week 8 postpartum	Mean change	95% CI of change	p value
	mean, SD	mean, SD			paired t-test
Sleep duration (h:mm)	7:19 (1:28)	6:30 (1:18)	−0:49	−0:53; −0:44	<.001
Sleep efficiency (%)	84.0 (14.0)	74.9 (13.9)	−9.19	−9.94; −8.42	<.001
Time in bed (h:mm)	8:44 (1:16)	8:47 (1:26)	0:03	−0:01; 0:07	.21
Time baby awake (h:mm)	-	1:53 (1:10)			-
**BIS. sum score**	**17.19 (10.3)**	**15.47 (8.9)**	**−1.72**	**−2.17; −1.27**	**<.001**
Sleep initiation	2.73 (2.4)	1.49 (2.0)	−1.24	−1.34; −1.13	<.001
Sleep maintenance	2.89 (2.3)	4.24 (2.8)	1.35	1.21; 1.49	<.001
Early awakening	2.30 (2.3)	1.29 (2.0)	−1.01	−1.12; −0.88	<.001
Non-restorative sleep	3.54 (2.2)	3.69 (2.3)	.15	−.26: −.03	.013
Daytime impairment	2.28 (2.2)	1.79 (2.0)	−.49	.38; .58	<.001
Satisfaction with sleep	3.46 (2.3)	2.98 (2.3)	−.48	.37; .60	<.001
	n (%)	n (%)			McNemars test
**Insomnia DSM-IV**	**1284 (61.6)**	**1126 (53.8)**	**−7.8%**	**−10.4; −5.2**	**<.001**
Criterion A Nighttime	1687 (80.8)	1786 (85.4)	4.6%	2.6; 6.6	<.001
Criterion B Daytime	1350 (64.8)	1150 (55.0)	−9.8%	−12.3; −7.2	<.001

BIS - Bergen Insomnia Scale - sub scores represent number of days per week with the insomnia symptom.

h:mm - hours:minutes.

Primiparous women reported significantly lower sleep efficiencies (3.8% difference; p<.001), and reported that their babies were awake significantly longer (26 minutes more; p<.001) during the night compared to babies of multiparous women (Table 2). They had significantly higher BIS scores (15.9 versus 14.9; p<0.001), mainly due to more problems with sleep-onset latency and night-time awakenings. This did not, however, result in a higher proportion of insomnia. Women who breastfed did not have significantly different BIS scores or prevalence of insomnia compared to women who were not breastfeeding. However, they reported fewer nights with sleep onset insomnia (BIS 1 sub score 1.4 versus 2.1; p<0.001) and more nights with wakefulness after sleep onset insomnia (BIS 2 sub score 4.3 versus 3.7; p = 0.003).

**Table 2:** Sleep after delivery, according to parity among 2088 women.

Sleep variable	Primipara	Multipara	Mean difference	95% CI	p-value
	n = 1058	n = 1026			
	mean (SD)	mean (SD)			t-test
Sleep duration (h:mm)	6:33 (1:24)	6:26 (01.11)	−0:07	−0:14; 0:00	.031
Sleep efficiency (%)	73.1 (14.6)	76.8 (12.8)	3.77	2.57; 4.96	<.001
Time in bed (h:mm)	9:07 (1:33)	8:26 (1:13)	−0:40	−0:47; −0:33	<.001
Time baby awake (h:mm)	2:07 (1:12)	1:41 (1:05)	−0:26	−0:32; −0:20	<.001
	mean (SD)	mean (SD)			
**BIS, sum**	**15.97 (9.1)**	**14.94 (8.8)**	**−1.03**	**−1.79; −.26**	**<.001**
Sleep initiation	1.74 (2.1)	1.23 (1.9)	−.51	−.67; −.32	<.001
Sleep maintenance	4.55 (2.7)	3.92 (2.8)	−.63	−.87; −.40	<.001
Early awakening	1.35 (2.1)	1.22 (2.0)	−.13	−.31; .04	.13
Non-restorative sleep	3.64 (2.3)	3.74 (2.3)	.10	−.10; .29	.34
Daytime impairment	1.74 (2.0)	1.84 (2.0)	.10	−.08; .27	.27
Satisfaction with sleep	2.95 (2.3)	3.00 (2.4)	.05	−.15; .25	.78
	n (%)	n (%)			Chi X^2^
**Insomnia DSM-IV**	**567 (53.3%)**	**556 (53.9%)**	**0.6%**	**−3.4; 4.7**	**.79**
Criterion A Nighttime	916 (86.1%)	866 (84.6%)	−1.5%	−4.4; 1.3	.17
Criterion B Daytime	579 (54.6%)	568 (55.1%)	0.5%	−3.5; 4.6	.79

BIS - Bergen Insomnia Scale - sub scores represent number of days per week with the insomnia symptom.

h:mm - hours:minutes.

Women who recovered from being depressed during pregnancy (EPDS ≥10 in pregnancy only) still had significantly higher insomnia (BIS) scores postpartum compared to women who scored low on the EPDS at both times (Table 3). Furthermore, table 3 shows that women who developed new depression (EPDS ≥10 postpartum only) had higher insomnia (BIS) scores in late pregnancy compared to women who remained well. Estimated sleep duration and sleep efficiency did not predict recovery from depression or later development of depression. Women who developed new depression postpartum had the largest negative change in mean sleep duration (−90 minutes) and sleep efficiency (−13.6%). The longer periods of wakefulness at night for postpartum women scoring 10 or more on the EPDS were mainly accounted for by the baby being awake more, as the differences between maternal and infant wake times were similar across the groups.

**Table 3:** Differences in insomnia scores and other sleep parameters according to depressive status+ before and after delivery among 2088 women.

	Not depressed+ pre or postpartum	New onset postpartum	Recovered postpartum	Depressed+ both pre and post
	n = 1657	n = 162	n = 153	n = 116
	Mean (SD)	Mean (SD)	Mean (SD)	Mean (SD)
**BIS, sum score**				
3rd trimester	15.8 (9.9)	18.9 (9.9)** a	22.7 (10.1)** a	26.2 (10.0)** ^b,^ * ^c^
Postpartum	14.0 (8.3)	22.1 (9.2)** a	17.8 (8.7)** a	23.8 (8.2)** ^c^
**Sleep duration (h:mm)**				
3rd trimester	7:22 (1:24)	7:22 (1:18)	7:11 (1:54)	6:50 (1:36)* ^b^
Postpartum	6:36 (1:18)	5:52 (1:18)** ^a^	6:32 (1:30)	5:56 (1:30)* ^c^
Baby wake time	1:50 (1:08)	2:23 (1:15)** ^a,^ c	1:50 (1:04)	2:15 (1:23)* ^a,^ c
Difference mum/baby	0:26	0:19	0:24	0:25
**Sleep efficiency**				
3rd trimester	84.8% (13.3)	84.2% (13.6)	79.3% (17.5)** ^a^	78.6% (15.9)* ^b^
Postpartum	75.5% (13.3)	70.6% (15.5)** ^a^	75.3% (14.1)	70.8% (17.4)* ^c^

+ Depression was defined by EPDS (Edinburgh Postnatal Depression Scale) score ≥10.

BIS - Bergen insomnia Scale SD: Standard Deviation.

h:mm - hour: minutes.

**p<.001.

*p<.01.

a- difference from women with no depression before or after delivery.

b- difference from women with new onset depression postpartum.

c- difference from women who recovered from depression in pregnancy.

Table 4 shows the results of a multivariate linear regression analysis of factors associated with higher insomnia (BIS) scores after delivery and factors associated with postpartum depression (EPDS scores). Insomnia scores during pregnancy were not a risk factor for depressive symptoms after delivery when adjusted for previous depression.

**Table 4:** Risk factors for insomnia and for depressive symptoms eight weeks after delivery, results from a multivariate linear regression analysis among 1914 women.

	Variable	B	Adj. B	P value	95% CI		p value
							Wilk's test*
**Insomnia** a	**Intercept**		**−4.30**	**.213**	**−11.08**	**2.48**	**.037**
(BIS score)	Primipara	.98	1.51	<.001	.74	2.29	<.001
after delivery	Previous depression	3.89	1.00	.018	.17	1.82	<.001
	Fear of delivery	5.72	2.23	.002	.82	3.63	.008
	Stressfactors last year	.67	.19	.11	−.04	.42	.001
	BIS week 32	.35	.30	<.001	.26	.33	<.001
	Log EPDS week 32	7.27	1.91	.008	.51	3.30	<.001
	Log HSCL-A week 32	12.27	3.09	.012	.67	5.51	<.001
	Education level	.33	.81	.023	.11	1.50	.020
	Maternal age	.01	.08	.058	−.003	.17	.16
**Depression** b	**Intercept**		**−.39**	**.001**	**−.63**	**−.15**	*
(log EPDS)	Primipara	.03	.04	.002	.02	.07	
after delivery	Previous depression	.22	.09	<.001	.06	.11	
	Fear of delivery	.23	.02	.35	−.03	.07	
	Stressfactors last year	.05	.02	<.001	.01	.02	
	BIS week 32	.008	.00	.66	−.001	.002	
	Log EPDS week 32	.58	.43	<.001	.38	.48	
	Log HSCL-A week 32	.80	.23	<.001	.14	.31	
	Education level	−.01	.03	.025	.004	.05	
	Maternal age	−.004	.00	.76	−.003	.004	

a R^2^ = 0.209.

b R^2^ = 0.356.

*p values for Wilk's Lambda for the significance of the multivariate tests (the p values are common for insomnia and depression).

CI - Confidence Interval.

BIS - Bergen Insomnia Scale.

EPDS - Edinburgh Postnatal Depression Scale.

HSCL A - Hopkins Symptoms Checklist, Anxiety module.

## Discussion

We found that women slept fewer hours at night after delivery compared to during late pregnancy, and that they reported more nights with night-time awakenings. Hence, sleep efficiency decreased from 84% to 75%. In spite of this, the self-reported insomnia scores improved, and the prevalence of insomnia according to the DSM-IV criteria decreased. Primiparous women reported lower sleep efficiency and higher insomnia scores than multiparous. Primiparous women also had increased risk of depressive symptoms after delivery. Insomnia scores in pregnancy, but not sleep duration or sleep efficiency, were associated with depressive symptoms postpartum. However, when adjusted for previous depression, insomnia in pregnancy did not predict depressive symptoms after delivery.

### Change in sleep from pregnancy to postpartum

We found that women slept fewer hours and less efficiently after delivery when compared to sleep during pregnancy. Montgomery-Downs et al. found longer sleep duration after delivery than in our study (7.2 hours compared to 6.5 hours) but similar low sleep efficiency [44]. In spite of reduced sleep time and sleep efficiency, insomnia scores and prevalence of insomnia were reduced in our study. Although they reported more nights of being awake for longer than 30 minutes, the postpartum women had fewer evenings with delayed sleep onset and fewer mornings with awakenings more than 30 minutes earlier than desired. Women reported less daytime impairment and were more satisfied with their sleep after delivery compared to during pregnancy. New mothers may have expected sleep disturbances after delivery to be more severe than they experienced, and therefore they were more satisfied with the (shorter amount of) sleep they got. In addition, waking up because of an infant may be more tolerable and may produce less worry compared to being unable to sleep for other reasons. Worry about one's sleep may contribute to insomnia, as individuals who tend to focus cognitively on their insomnia and ruminate about their poor sleep are less likely to get a good night's sleep [1]. This worry may consequently lead to hyper-arousal, an important factor in maintaining insomnia [45]. None of the women were working outside the home at the time of the postpartum follow-up, due to the right of paid maternity leave in Norway for the first 45 postnatal weeks. This may explain some of the lower insomnia rate, as sleep problems are more common during periods of active employment [46]. However, the majority of the women in our study, 63%, were on sick leave in week 32, and these women had a higher rate of insomnia compared to working pregnant women [47]. Taking care of a new-born baby the first weeks after delivery may be hard work and not considered leisure time. However, compared to work outside the home, there may be more flexibility in terms of completing tasks and more opportunities for daytime napping. Our study did not measure daytime napping, as this is not part of the Bergen Insomnia Scale or the Pittsburgh Sleep Quality Index, but a study of pregnant women in Taiwan found decreased sleep among women with longer work hours and women who had longer daytime naps [48]. Future studies should include questions regarding daytime naps.

Primiparous women reported more days with delayed sleep-onset time and were awake during more nights compared to multiparous women, possibly due to less experience and confidence in child care and more worry and hyper-arousal. Coo Calcagni et al. found that first-time mothers experienced more stress and had poorer subjective sleep than multiparous women [49]. Other studies have also found poorer sleep among first-time mothers [13], [50]. Salo et al. found that difficulties initiating or maintaining sleep increased the long-term risk of depression in the general population [9]. These sleep factors, more prevalent among primipara, may be mediators that also increase the risk of postpartum depression. However, primiparous women also had an increased risk of depression when adjusting for level of insomnia, so there may be factors other than insomnia contributing to this increased risk for depression among first-time mothers. Previous studies from Norway have found conflicting results regarding parity and postpartum depression [13], [38], [51]. Parity does not seem to be among the strongest risk factors for postpartum depression [25]. We found no associations between breastfeeding and total insomnia scores, which is in line with other studies [13], [52]. From our data, it seems that although breastfeeding may increase the number of nights with awakenings, it also decreases nights with prolonged sleep-onset latency.

### Insomnia and depression

We found that insomnia, but not sleep efficiency or sleep duration, was a risk factor for postnatal depression in the univariate analyses. This is similar to findings by Park et al., using actigraphy and subjective sleep reports. They found that subjective assessments of sleep may be more accurate predictors of postpartum depression than total amount of sleep measured by actigraphy [53]. In our study, women who recovered from antepartum depression still had residual insomnia symptoms compared to women who scored low for depression at both time points. Similarly, women who developed depression after delivery but were not depressed in pregnancy had higher insomnia scores in pregnancy compared to women who remained well. This may reflect the co-morbid and bidirectional relationship between insomnia and depression [10], [54]. It may also reflect a pre-clinical state where women have insomnia as a precursor for or an early symptom of depression.

The residual insomnia found among women who recovered from depression in pregnancy has previously been reported in non-pregnant populations and is associated with an increased risk of relapse of depression [10], [55]. This may explain why insomnia in pregnancy did not predict postpartum depression when adjusted for previous depression. Antenatal depression and a prior history of depression are among the strongest risk factors for postnatal depression [56]. Marques et al. found similar results to our study: Insomnia in pregnancy seemed to predict depression after delivery; however, when adjusted for lifetime history of depression and negative affect in pregnancy, the association disappeared [14]. In a study of women with previous postpartum depression, Okun et al. found that changes in postpartum sleep during the first 17 weeks predicted recurrence of depression in these at-risk women [28]. Insomnia in pregnancy may thus be seen as a mediator between previous depression and a new depressive episode postpartum for women at risk. As insomnia is an easy complaint to look for during pregnancy, insomnia may thus warn the clinician of an increased risk of postpartum relapse of depression. This may in turn provide opportunities for early interventions to prevent clinical depression.

Treating insomnia during pregnancy and the early postpartum period for women with previous depression may be a way of preventing postpartum depression. One pilot study of insomnia treatment among 12 women with postpartum depression and insomnia showed promising results on sleep efficiency, total wake time, subjective mood, insomnia severity, sleep quality, and fatigue [57]; however, more studies on treatment of postpartum women are needed. An encouraging finding of our study is that women with insomnia in pregnancy who have never been depressed before may be reassured that insomnia in itself does not seem to increase the risk for postpartum depression.

For many, insomnia is a chronic condition. The present study found that a woman who slept poorly during pregnancy was more likely to sleep poorly after delivery as well, regardless of any depression. Morin and colleagues found that 74% of people with insomnia had the complaint for more than one year, and more than 25% of those who recovered experienced relapse [58]. The perinatal period may therefore be no exception to this. Hyper-arousal may be one factor explaining that both anxiety, as measured by the HCSL-Anxiety scale, and fear of delivery were factors associated with postpartum insomnia [45]. There may be an interaction between cognitive and autonomic hyper-arousal that contributes to the maintenance of both insomnia and continued emotional disturbance [59], [60].

### Strengths and limitations

This is a large study from a general population of pregnant women. However, as women who did not understand the Norwegian language were excluded, these results may not be representative for the newer immigrant population. The longitudinal design makes it possible to examine the direction of effects and to evaluate whether depressive symptoms precede insomnia or whether insomnia is a precursor for depression in this period with decreased sleep among all women. For longitudinal population-based studies, a retain rate of more than 50% after three questionnaires is acceptable, and a large number of participants remained in the study. According to Galea and Tracey, declines in participation rates are not likely to have substantial influence on exposure-disease associations [61]. Women with depressive symptoms during pregnancy were less likely to remain in the study, and this may have led to selection bias. We found, however, a strong correlation between depression and insomnia in our sample with possibly fewer depressed women than in the population, and the real relationship may be stronger than the results presented here. The questionnaires included several known risk factors for postpartum depression, making it possible to adjust for confounders. We controlled for stressful life events the previous year, but did not assess for past life events, such as childhood adversities, which may also be of importance for emotional dysfunction during pregnancy [62]. The tools used are all validated and have been used in several other studies in Norway. Depression and insomnia were measured by self-report and not verified by clinical interviews or sleep diaries. As the diagnosis of insomnia relies on the subjective experiences of the individual experiencing sleep problems [5], self-report may be an adequate way of measuring insomnia. The BIS is based on recall of insomnia symptoms during the past month, and the prospective use of sleep diaries might have given a more accurate estimate of current insomnia. However, BIS scores correspond well with polysomnographic sleep registration, except for item 4 (feeling adequately rested after sleep) and item 5 (daytime functioning) [29]. The EPDS is not diagnostic of depression, and a cut-off at 9/10 at the Norwegian version is relatively low compared to validations in other languages [63]. There may therefore be a risk of some false positives in Table 3. However, the continuous scale of the EPDS has been recommended for use in population research to find factors associated with depression [64]; hence the cut-off on the EPDS did not influence the risk factors identified in Table 4.

### Suggestions for clinical intervention and further research

Insomnia during pregnancy may be a valid clinical marker for recurrence of depression postpartum among women at risk. Further studies should evaluate whether treatment of insomnia during pregnancy may prevent postpartum depression among women with other risk factors for the condition. Cognitive behavioral therapy for insomnia may have a positive effect on anxiety [65], and treatment of depression with mindfulness meditation improves subjective sleep quality with corresponding changes in polysomnography [66]. Lee and Gay found that treatment of sleep problems among new parents using a modified sleep-hygiene intervention improved sleep, especially among less socioeconomically advantaged women [67]. Low socioeconomic status is a risk factor for postpartum depression [25], and improving sleep for these mothers may therefore be one way of preventing or relieving depressive symptoms in this group.
